# A review of the handling of missing longitudinal outcome data in clinical trials

**DOI:** 10.1186/1745-6215-15-237

**Published:** 2014-06-19

**Authors:** Matthew Powney, Paula Williamson, Jamie Kirkham, Ruwanthi Kolamunnage-Dona

**Affiliations:** 1 Institute of Translational Medicine, University of Liverpool, Crown Street, L69 3GS Liverpool, UK

**Keywords:** Review, Missing, Data, Handling, Longitudinal, Repeated, Measures

## Abstract

The aim of this review was to establish the frequency with which trials take into account missingness, and to discover what methods trialists use for adjustment in randomised controlled trials with longitudinal measurements. Failing to address the problems that can arise from missing outcome data can result in misleading conclusions. Missing data should be addressed as a means of a sensitivity analysis of the complete case analysis results. One hundred publications of randomised controlled trials with longitudinal measurements were selected randomly from trial publications from the years 2005 to 2012. Information was extracted from these trials, including whether reasons for dropout were reported, what methods were used for handing the missing data, whether there was any explanation of the methods for missing data handling, and whether a statistician was involved in the analysis. The main focus of the review was on missing data post dropout rather than missing interim data. Of all the papers in the study, 9 (9%) had no missing data. More than half of the papers included in the study failed to make any attempt to explain the reasons for their choice of missing data handling method. Of the papers with clear missing data handling methods, 44 papers (50%) used adequate methods of missing data handling, whereas 30 (34%) of the papers used missing data methods which may not have been appropriate. In the remaining 17 papers (19%), it was difficult to assess the validity of the methods used. An imputation method was used in 18 papers (20%). Multiple imputation methods were introduced in 1987 and are an efficient way of accounting for missing data in general, and yet only 4 papers used these methods. Out of the 18 papers which used imputation, only 7 displayed the results as a sensitivity analysis of the complete case analysis results. 61% of the papers that used an imputation explained the reasons for their chosen method. Just under a third of the papers made no reference to reasons for missing outcome data. There was little consistency in reporting of missing data within longitudinal trials.

## Review

In clinical studies in general, missing data is a problem that frequently arises [1]. In any given trial, measures are taken to assure that the level of missingness is as low as possible, as high percentages of missing data can prove problematic in establishing the true clinical effectiveness of different treatments [2]. It can occasionally be difficult to pinpoint the reasons for missingness after the clinical elements of a trial have terminated, so there is a benefit to monitoring the reasons for missing data while the trial is ongoing. The most accurate estimates of treatment effects within a trial can be obtained if we take into account the patients with missing data values, as well as those who completed the trial with a full set of outcome data [3]. In order to account for this missing data in the most appropriate manner, it is essential to record the reasons for missingness and take these reasons into account when selecting an appropriate method of statistical analysis [4].

Missing outcome data in longitudinal and repeated measures studies can prove particularly problematic, as organising the collection of multiple data points per patient can result in higher percentages of missingness. However, one advantage we have for dealing with the missing data in longitudinal studies over single reading studies is that we can use previous readings of thelongitudinal outcome to gain more accurate estimates of the unknown outcomes. Many methods for missing data handling have been introduced in the past 30 years including modelling techniques which attempt to account for missing data, such as mixed models and joint modelling of longitudinal and time-to-event data, and imputation methods. With these modern developments it is of interest to observe how missing data has been dealt with in recent clinical trials [5-8].

By reviewing all trials published between July and September of 2001 in four leading journals, White, Wood and Thompson came to the conclusion that missing outcome data was a large problem in clinical trials and that missing data was ‘often inadequately handled’. Observing the results from this study, 17 out of the 37 (46%) of the repeated measures clinical trials analysed used a complete case analysis method for dealing with the missing data, which excludes patients with any missing values [3]. The majority of papers included in this review did not have repeated measurements recorded over time. Little research has been done into how missing data is handled specifically in clinical trials with longitudinal outcome data in practice.

One method of dealing with missing data in clinical trials is imputation. Imputation methods can be categorised as either simple or multiple. Simple methods involve the imputation of one value and multiple imputation techniques can be used to generate multiple imputed datasets, the results of which are then pooled to provide estimates of treatment effect. A poor choice of simple imputation mechanism can lead to incorrect conclusions about treatment effect [9]. When using a simple imputation, Rubin [6] was concerned that, while these methods were easy to employ, they failed to preserve the variability in the dataset. Multiple imputations are a more flexible method, as they take into account a greater range of missing data possibilities and also address the issue of variability [10].

In the aforementioned review by Wood et al., the most commonly used method of imputation was Last Observation Carried Forward (LOCF), which was used in 5 out of 37 (13.5%) of the papers [3]. Alternative simple imputation methods were used in 6 out of 37 (16%), while only 1 out of 37 (2.7%) of the papers with longitudinal readings in this study used multiple imputation methods.

Establishing the reasons for missingness within a trial and using appropriate methods based on this information is of utmost importance. An appropriate strategy for imputation within a trial with justification can be put into action, and the results from this imputed dataset can then be compared with the results of a complete case analysis. This would allow the investigator to assess both analyses on their own merits, and draw conclusions from both estimates. Different conclusions when comparing simple imputation to the complete cases indicate the data is not Missing Completely at Random, and similarly different conclusions with the multiply imputed and complete case datasets suggest data is Not Missing at Random [9].

As we understand that the need to use as much information as possible about missingness within trials is such an important issue, this will be the main focus of the paper. Unlike previously published systematic reviews that focus on missing data, this paper exclusively includes trials which have some form of longitudinal measurements taken. The review here does not select from specific journals based on their impact factor in order to provide the most general results possible within the framework of randomised controlled trials (RCTs) with longitudinal measurements.

## Methods

The review was conducted in order to gain information about the frequency and extent to which missing data was recognised as an issue in trials with longitudinal measurements, and how it was dealt with. We aimed to establish how often these trialists collect the reasons for missing data and whether these are reported in the analysis. In particular, we aimed to ascertain details about the use of imputation in these trials; whether imputation was used, and if an explanation for doing so was provided in the text. One thing which could dictate the need for imputation in such a study is the percentage of completing patients, as low percentages of missing data will yield similar conclusions in an imputed dataset as a complete case analysis [2]. In such a case, statisticians or clinicians may be justified in claiming that missing data methods such as imputation are not required. We investigated to establish if there was a relationship between the use of imputation methods and the percentage of completing patients. Also, it seems intuitive that papers which have had the benefit of a statistician’s knowledge of these techniques would have a more comprehensive analysis and acknowledgement of the problems provided by missing data; so we aimed to establish whether there was evidence to support this.

The development of imputation techniques is the subject of ongoing research within the statistics community. In this study we wanted to investigate whether there had been a rise in the use of these methods in the past few years for studies where dropout occurred, and also whether these imputed results were presented alongside the complete case analysis results, as this level of detail can prove useful to a clinician.

In this study, dropout is defined as a patient having no more longitudinal readings until the end of the follow-up period. While the missing data for patients who have not dropped out is also an issue which can be dealt with in largely the same way as that for dropouts, the main focus of our extracted data will be on those individuals who withdrew from each trial.

### Eligibility criteria

We included papers that were described as a ‘randomised control trial’ with longitudinal or repeated measurements taken at some point in the trial. The longitudinal measurements also had to be balanced; this can be defined as having measurements recorded at the same set time points for each patient. As we were assessing the recent use of methods and recent attitudes to missing data in longitudinal studies, we only included papers that were published from the years 2005 to 2012, and no restrictions were put on journal.

All papers that were not written in English or had non-human participants as the subjects were excluded from the study, as well as any papers with only binary outcomes being recorded longitudinally.

### Study selection and data extraction

From all the papers identified as eligible for this study, 100 were selected at random due to time constraints. This randomisation was done by ordering the papers alphabetically by first author surname, giving each of these papers a random number, and then randomly generating a sequence of the integers using the ‘random’ function in the R statistical software. The data was then extracted from each paper in order. If a paper was found to be ineligible on closer inspection, then the 101st paper in the sequence was added to the study, and then the 102nd and so on. If less than 100 eligible papers were identified, then all papers were included. To identify potential papers for inclusion, MEDLINE (Ovid interface) was searched using the following terms; longitudinal randomi$ed controlled trial$ or repeated measure$ randomi$ed controlled trial$ or longitudinal RCT$ or the same searches with ‘controlled’ replaced by ‘control’. The papers identified also had to fall within the constraints of our prespecified eligibility criteria.

An extraction form designed to collect all necessary information was created and verified by the authors listed within the study. The 100 papers had data extracted by the first author (MP). The summary of the extracted data was reviewed by the second and third authors (RKD, JK). If there were any ambiguities or confusion as to the extracted data, the second and third authors were consulted.

For each longitudinal trial, data was extracted relating to the general characteristics of the trial, as well as more specifically the details relating to missing data handling. Our main focus was to look at the missing data handling methods used for patients that dropped out, as opposed to just those with missing interim values. Details of the nature of the longitudinal data, how many time points there were and whether a primary longitudinal outcome was recorded, were extracted. We were interested in the percentage of completing patients within the study, as we felt that there may be a greater need for imputation-based methods with larger amounts of missing data, and details of this were extracted from each trial. The imputation method used in each study was recorded, as well as the level of explanation for using the chosen method for missing data handling and whether each trial recorded reasons for dropout within a study. We also provided an assessment of whether we felt that the methods for missing data handling were appropriate in each paper. Whether a statistician was present as one of the authors of the study, as well as the software used were also noted. Finally, for the papers that used imputation we collected information about whether a comparison was made between the complete case and imputed datasets, as well as whether these analyses yielded different statistical conclusions.

### Data analysis

Counts were made on the number of papers that fall into each category, as well as cross references between the different abstracted groups to search for potential correlations.

## Results

**Figure 1:**
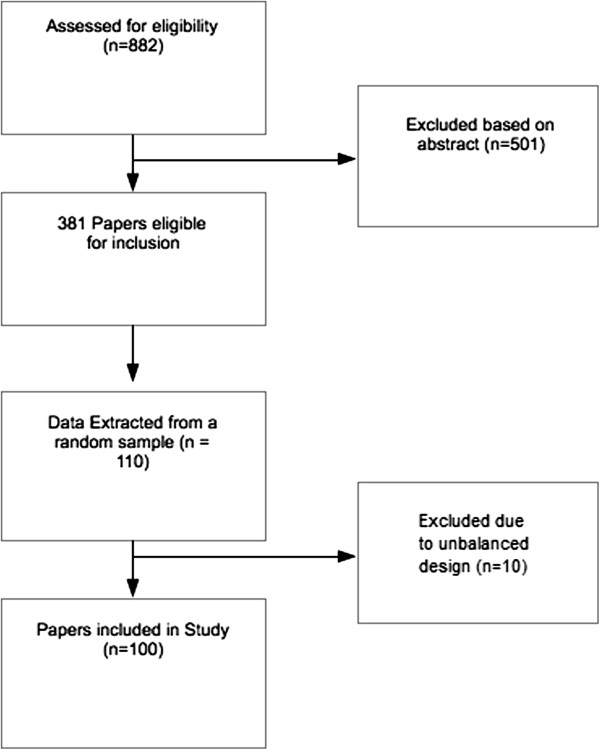
**CONSORT diagram of the systematic review process.** A CONSORT diagram describing the process of how many papers were eligible for inclusion and how many were eliminated after our initial search.

Potential papers for inclusion were identified using the MEDLINE (Ovid interface). A CONSORT diagram of the progress is included as Figure 1.

A total of 882 hits were obtained from the search strategy. These 882 abstracts were screened for potentially eligible papers, which narrowed down to 381 papers eligible for inclusion. After a randomisation of the order of these papers, 10 of the papers we attempted to extract data from were found to be ineligible due to the unbalanced nature of their longitudinal readings. Therefore, we read through 110 papers before establishing 100 which were eligible. A full list of the eligible papers included in the study are provided in Additional file 1.

### Methods of imputation

Data was collected from papers from a wide range of different medical areas in order to investigate how missing data was handled in practice for randomised control trials with longitudinal measurements. The most popular medical areas were mental health (13%), cancer (11%) and rheumatology (10%). Greater detail of the properties of these trials is provided in Table 1, and Table 2 lists the primary method of imputation for missing data handling within the study. Table 3 provides a full list of medical areas included in the study.

**Table 1:** Method of missing data handling

**Primary approach to analysis**	**Papers**
Complete case analysis	32
Mixed models	18
Simple imputation	14^1^
*LOCF/FOCB/Baseline Carried Forward*	*9*
*Average value either side Imputed*	*1*
*Simple algorithmic-based imputation*	*1*
*Mean of other patients values imputed*	*1*
*Median values imputed*	*1*
Multiple imputation methods	4
Other non-imputation-based methods^2^	14
Exclusion based on amounts of missingness	6
Exclusion based on reasons for missingness	1
No missing data	9
Unclear	3

^1^One paper which used a complete case analysis also used simple imputation as a secondary analysis. In Table 2, this paper is included in the simple imputation section.

^2^Comparison of means, for example, t-test, RMANOVA.

**Table 2:** Trial characteristics

			**Method of handling missing data**							**Acceptable method?**^**1**^		
		**No. of papers**	**Complete cases**	**Simple**^**2**^	**Multiple**	**Mixed models**	**No missing data**	**Unclear**	**Other**^**3**^	**Yes**	**No**	**Unclear**
Number of patients	1-100	48	17	8	0	4	8	3	8	18	9	13
	101-200	22	8	2	2	5	1	0	4	9	11	1
	201-300	12	4	2	0	4	0	0	2	8	4	0
	301-400	7	1	1	1	1	0	0	3	3	3	1
	400+	11	1	1	1	4	0	0	4	6	3	2
Country of publication	USA	53	20	7	2	10	4	0	10	20	18	11
	UK	40	8	6	2	7	3	3	11	20	12	5
	Denmark	3	2	0	0	0	1	0	0	2	0	0
	Netherlands	3	1	1	0	1	0	0	0	2	0	1
	Japan	1	0	0	0	0	1	0	0	0	0	0
Number of time points	3	38	13	4	3	10	1	1	6	14	16	7
	4	28	9	4	1	4	2	2	6	14	4	8
	5	16	4	3	0	3	2	0	4	10	3	1
	6	6	2	0	0	0	2	0	2	1	3	0
	7	4	1	0	0	0	2	0	1	1	1	0
	8+	8	2	3	0	1	0	0	2	4	3	1
Year	2005-06	19	9	2	0	1	2	1	4	7	4	6
	2007-08	27	8	3	0	5	4	0	7	11	10	2
	2009-10	25	7	3	1	4	3	1	6	9	8	5
	2011-12	29	7	6	3	8	0	1	4	17	8	4
Clinical area	Mental health	13	2	4	2	1	1	1	2	7	2	3
	Cancer	11	4	1	1	4	0	1	0	5	4	2
	Rheumatology	10	4	1	0	2	1	0	2	3	5	1
	Infectious diseases	8	4	1	0	1	1	0	1	4	2	1
	Heart and circulation	7	1	2	0	1	0	0	3	3	2	2
	Dentistry/oral health	6	3	1	0	0	2	0	0	3	1	0
	Neurology	6	2	0	0	2	0	0	2	2	2	2
	Anaesthesia and pain	6	3	0	0	1	0	0	2	2	2	2
	Other^4^	33	8	4	1	6	4	1	9	15	10	4
Dropout reasons recorded?	Yes	35 (39.8%)^5^	15	7	0	7	NA	NA	6	20	11	4
	Partial information	25 (28.4%)	7	5	1	7	NA	NA	5	15	9	1
	No	28 (31.8%)	10	2	3	4	NA	NA	9	9	10	9

^1^Based on the 91 papers which had missing data.

^2^One paper which used a complete case analysis also used simple imputation as a secondary analysis. In Table 2, this paper is included in the simple imputation section.

^3^Comparison of means e.g t-test, RMANOVA.

^4^A full list of medical areas is included in Table 3.

^5^Disregarding the 9 papers without missing data and 3 papers where the missing data handling method was unclear.

**Table 3:** Complete list of medical areas

**Medical area**	**Papers**
Mental health	13
Cancer	11
Rheumatology	10
Infectious diseases	8
Heart and circulation	7
Dentistry/oral health	6
Neurology	6
Anaesthesia and pain control	6
Blood disorders	3
Developmental, psychosocial, and learning problems	2
Endocrine and metabolic	5
Eye and vision	2
Gastroenterology	1
Health care of older people	2
Kidney disease	2
Lungs and airways	2
Neonatal care	2
Orthopaedics and trauma	4
Pregnancy and childbirth	3
Skin	1
Urology	1
Wounds	3

In Table 1, ‘Complete case analysis’ denotes the trials in which only patients that did not drop out were recorded. Those papers which would come under the category of ‘Mixed models’ are trials which took into account all available data, but used no imputation methods, as in many cases this type of analysis is sufficient for handling those patients that dropped out. Any papers which did not use imputation, did not exclude any data but did not used mixed models fall into the category of ‘Other non-imputation based methods’. We have listed how many papers used simple imputation and multiple imputation, with specific details of the nature of simple imputation methods underneath this heading.

As presented in Table 1, 9 papers had no missing data in their trials, and the 3 papers in the ‘Unclear’ category made no reference to missing data, and the analysis failed to mention any missingness or clarify whether or not imputation methods were used. Out of the 100 papers, 18 had used imputation and only 4 had used multiple imputation as the most advanced missing data handling method. One paper carried out a complete case analysis as the primary method of missing data handling, but included an analysis based on LOCF as a secondary method. For the papers that only included the complete cases, the most common methods of analysis were variations of ANOVA or ANCOVA in 13 trials, mixed modelling in 6 trials, t-tests for mean comparison in 5 trials and linear regression modelling in 4 trials.

The most common method of simple imputation used was LOCF or a variation of this method. LOCF was used in 8 papers. The clinical topics in these papers were quality of life (QoL) based on moral support in patients with depression, chronic muscle-based neck pain, chronic arm pain due to repetitive use, shoulder pain in stroke patients, outcomes in chronic obstructive pulmonary disease (COPD), stress levels in arthritic patients, amount of sleep in patients with chronic insomnia and number of behavioural disturbances in patients with dementia. In one trial, the baseline value was carried forward to impute outcome data at two and six months. LOCF may be a reasonable method if patients are in a steady state closer to the time of dropout, therefore the use of LOCF may be questionable in some of these disease areas such as stroke and dementia. For those papers that used simple imputation, as a primary method of analysis 5 trials used t-tests for comparison of means, 5 used linear mixed models, 3 used a variation of ANOVA and 1 used the chi-squared test. Of the trials that used multiple imputation methods, 2 used mixed modelling, 1 used linear regression modelling and 1 used t-tests as the primary method of analysis.

For the remaining results tables, we have excluded the papers with no missing data and the papers where the methods were unclear.

### Explanation of the reasons for using the statistical methods for handling missing data

When extracting data with regard to including some explanation of the statistical methods used for analysing the missing data, we found that 37 (42.0%) papers with missing values made comments on why they had used their particular method of choice. The level of explanation ranged from one-line statements about the efficiency of the chosen method, to multiple page descriptions of different missing data methods and the merits of each.

Of the papers with data extracted, 51 (58.0%) provided no explanation of the reasons for the missing data methods used. All 4 papers that employed multiple imputation methods provided an explanation of the reasons for their use. Out of the 14 papers that used simple imputation methods, 7 (50%) explained the reasons for their choice of imputation. A total of 26 out of 70 (37.1%) papers without imputation attempted to explain the reasons for the missing data handling method chosen. For the papers with no missing data, 1 of the 9 (11.1%) discussed missing data methods within the report, despite no missing data being presented when the trial was carried out. This was done with a view to suggesting how they were going to analyse the missing data should this issue arise.

### Was a statistician involved in the analysis?

It was found that out of the 88 papers with missing data, 30 (34.1%) had a statistician cited as one of the co-authors of the study. Results indicated that there was little difference in the levels of explanation of the missing data methods used when a statistician was co-authoring a paper. Out of the 37 papers which justified their missing data methods, 13 (35.1%) had a statistician present, compared to 17 out of 51 (33.3%) papers which failed to explain the reasons for their chosen method.

### Number of publications by year

**Figure 2:**
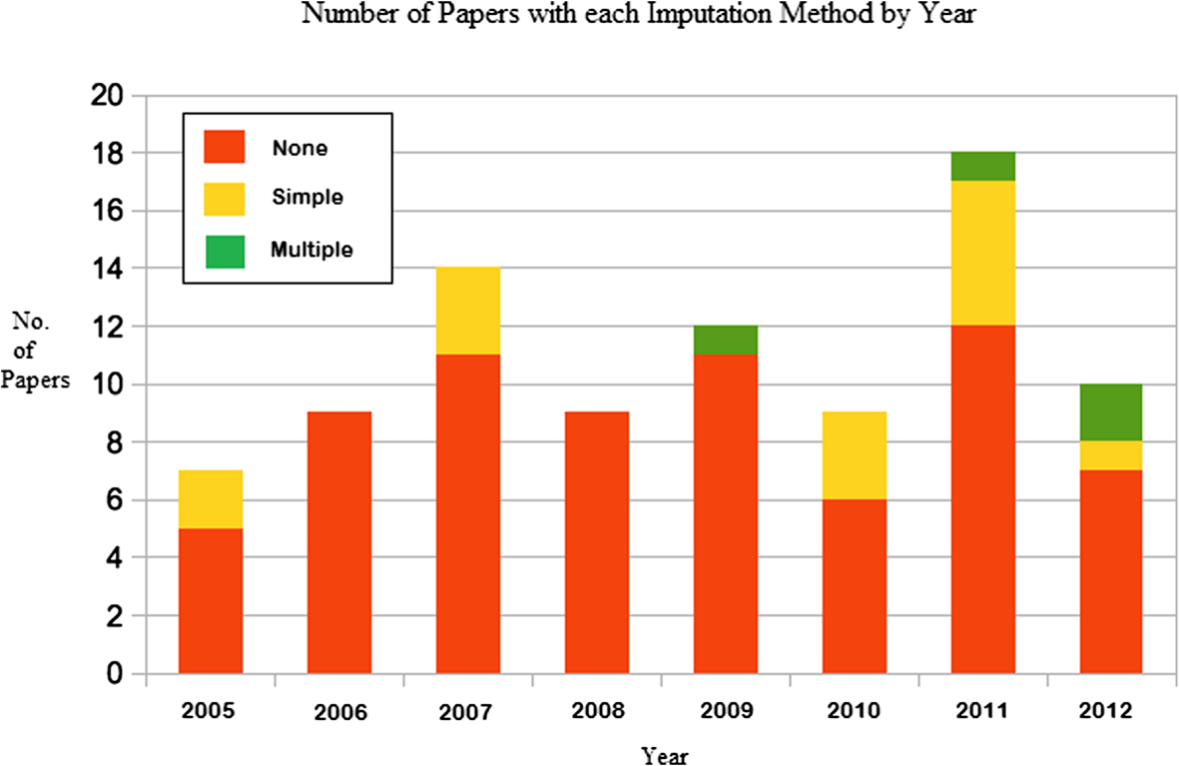
**Number of publications for each imputation method by year.** A graph displaying the number of publications which used the different types of imputation method each year.

We aimed to assess whether a greater number of papers had used imputation in recent years. Figure 2 displays these results.

All multiple imputation analysed papers were from 2009 onwards, and the majority of simple imputation based papers were published in the last three years. This could indicate an increase in a recognition and awareness of the benefits of using imputation within the past few years.

When we assessed whether the levels of explanation given for using the chosen missing data method had increased in the past few years, as well as the amount of reasons for missing data reporting, we found that there was not a substantial increase.

### The imputation method used, based on the percentage of completing patients within the study

**Figure 3:**
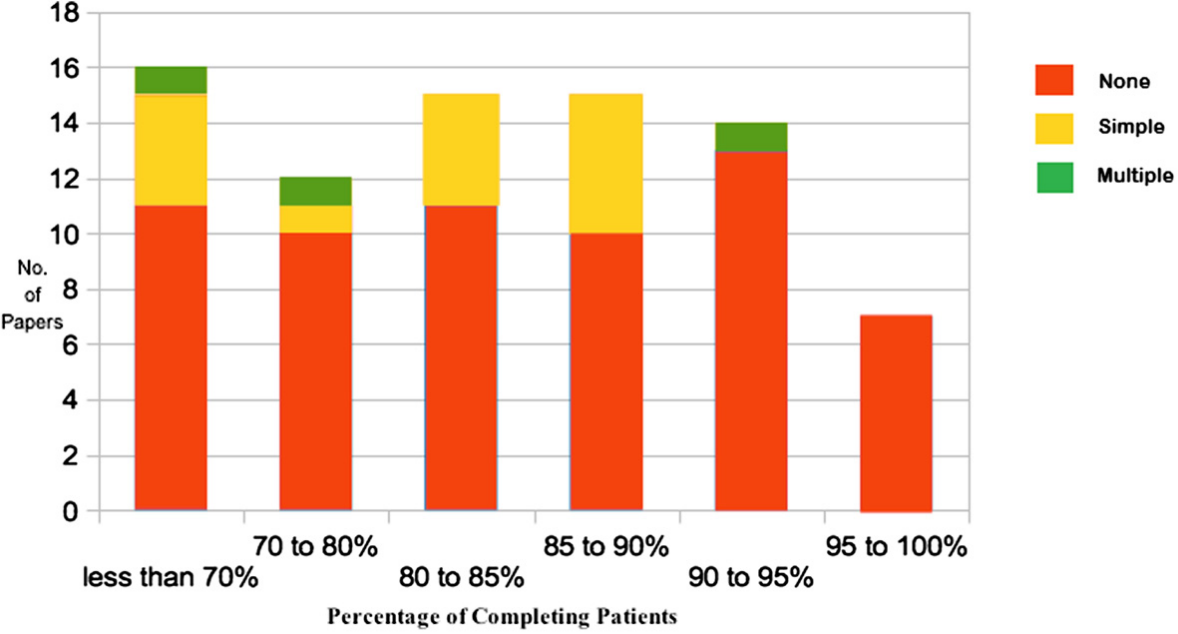
**Number of publications for each imputation method by percentage of completing patients.** A graph displaying the number of publications which used the different types of imputation based on the percentage of patients who completed their measurement schedule within the trial.

With larger percentages of missing data, there is a greater potential for bias if these non-completing patients are ignored within the analysis. We present Figure 3 to represent each missingness method based on the percentage of completing patients.

Out of the papers with a clear definition of the methods for handling missing data, 12 (12.3%) did not mention the percentage of completing patients. 11 (68.8%) of papers with less than 70% completing patients used no imputation methods, which could potentially lead to biased results. In general, trials with less than 10% patients dropping out rarely used imputation methods.

### Were the reasons for dropout recorded?

We assessed the level of information given for dropout in each study by putting each paper into one of the following categories. ‘Yes’ is defined as detailed discussion on missingness or reason for dropout, including a record of the number of people that dropped out and the specific reasons for dropout recorded at each time point. ‘Partial information’ is defined as less detailed with some mention of the reasons for dropout or missingness, but not necessarily indicating the number of patients at each time point or providing specific medical reasons. Papers in the ‘No’ category provided no details of the reasons for missing data.

We can see from Table 2 that 35 (39.8%) of papers fell into the ‘Yes’ category, providing substantial and detailed reasons for the missing data of each patient. In 25 (28.4%) of cases, the details of reasons for dropout could be categorised as ‘Partial information’. 28 (31.8%) of papers failed to provide any reasons for patients dropping out.

Where no reasons were recorded for missing data, 7 out of the 28 (25.0%) papers made an attempt to explain the reasons for the missing data handling method that was used. For the papers that had some reference to reasons for dropout, 30 out of 60 (50%) made an attempt to explain the reasons for their chosen method of missing data handling. However, the majority of these papers provided justification in terms of the general statistical benefits of the method used, as opposed to information relating to the specific prognostic factors and potential dropout reasons unique to the trial in question.

### Assessment of the appropriateness of the missing data methods used

We looked at the reasons given for the use of each missing data method within the 100 papers, and assessed whether we felt that the reasons given provided an adequate justification for that method.

Of the 37 papers that attempted to explain the reasons for their chosen missing data method, we felt that 19 (51.3%) had provided sufficient detail and justification for their choice. For 5 papers, it was unclear whether the justifications provided were enough, and in 13 (35.1%) of the cases we felt that the justification provided was insufficient for the method used. In many of these cases, the author provided reasons which may have been applicable in certain situations, but failed to acknowledge the specifics of the trial in question, for example, ‘certain relatively simple methods can be appropriate’ referring to a complete case analysis when the trial in question has a high percentage of missing outcome data. While this justification may be appropriate in some trials with low percentages of missing data despite the vagueness of the explanation, it disregards the fact that the trial in question had over 30% missing data.

In Table 2, we also provided our own assessment as to whether the methods used were appropriate, when possible to determine, in each paper. In 44 (50%) of the papers with missing data where the methods were clear, we felt that the method used to handle missing data was appropriate, for example, the LOCF method for missing data being used in a trial where there was a steady state outcome. It was difficult to determine the appropriateness in 14 (15.9%) of the papers, for example, in trials where the amount of missing data was not recorded in the paper and either a complete case or mixed model analysis was used. In 30 (34.1%) of the papers we felt that the method used was not appropriate, for example, in a trial with high percentages of missing data that used a complete case analysis.

We judged that in all cases where multiple imputation was used, this was an appropriate method, and only in 2 papers out of 14 (14.3%) was simple imputation deemed inappropriate. There were 13 (40.6%) which used a complete case analysis where the percentage of missing data was too high (over 10%) to justify. We concluded that the majority of papers which used mixed models without imputation as a form of missing data handling were justified in doing so.

### Imputed datasets as a comparison to non-imputed data

Out of the 18 papers that used imputation methods, 11 (61.1%) made no reference to a comparison between the complete case analysis results and the imputed dataset results. Of the 7 papers that made the comparison, 2 (28.6%) of them yielded different clinical conclusions about treatment effect when complete case analysis was compared to one of the imputed datasets, although one paper only provided the details of this difference for the purpose of illustration. One of the 2 papers (mental health) had 43.2% dropout, and reported the P-values for treatment comparison using the complete case (P = 0.428), mean imputation (P = 0.360), LOCF (P = 0.026), and multiple imputation (P = 0.426). The treatment effect was significant when LOCF was used but not when other imputation methods were used. However, in this case the authors suggested that LOCF was not an appropriate method to use for missing outcome data in their trial, and therefore concluded no difference in treatment effect. This illustrates how an incorrect choice of missing data handling method can influence the results, and in this particular case the authors used an appropriate method in order to gain accurate results. The second study, also in mental health, stated that there had been a difference in conclusion without presenting both sets of results. Both of these papers had less than 70% of patients completing the study.

## Discussion

### The extent of missing data handling and use of imputation methods

In the CONSORT statement, point 13b. states "for each group, losses and exclusions after randomisation, together with reasons" should be included within the trial report [11]. This was not adhered to in a large number of trials within the study. It is difficult to suggest a gold standard for missing data handling, as the appropriateness of a method is dependent on the unique nature of missing data within each individual trial. However, by carrying out a complete case analysis or eliminating certain patients based on level of missingness or prognostic factor we are making a bold assumption that the data we exclude is missing completely at random. This is rarely, if ever, the case in practice [9]. Therefore, it was disappointing to see that 39 out of 88 (44.3*%*) papers that had patients with missing data excluded records. The 32 out of 88 (36.4%) papers that carried out a complete case analysis indicates a decrease in the use of this method since the study by Wood, White and Thompson in 2004 [3]. A greater understanding of the benefits and methods of multiple imputation has been developed in recent years. However, only 4 papers within the study used multiple imputation, with the evidence suggesting that statisticians are more frequently using simple imputation methods. One positive sign is that the trends suggest that more papers in the past few years have been using multiple imputation methods. It was also interesting to discover that having a statistician involved within a trial investigation did not appear to have much of an impact on the choice of imputation method or the level of explanation for such a method being used.

As well as the aim of obtaining an accurate estimate of clinical outcome, there is a lot to be said for establishing the properties of the missing data. The goal of imputation is to create a full dataset with similar properties to that which would have been observed had no missing data been present. The use of some simple imputation methods, such as best/worst case value imputation may provide us with more extreme results, with a potential for biased estimates. In many cases, the use of simple imputation methods without a detailed explanation of reasons can raise as many questions as they answer, and careful justification should be provided in order to demonstrate their validity in each individual trial. Within the trials analysed in this study, half the papers that used simple imputation within the study justified their reasons for use.

One factor which is prominent in establishing whether imputation methods may be beneficial is looking at the amount of missing data and the percentage of patients dropping out of a trial. The findings of the paper published by White, Wood and Thompson showed that imputation was more frequently used in papers with larger amounts of missing data. In this study, while papers with a lower percentage of dropouts appeared to be less frequent in their use of imputation, there was still a large number of papers with high levels of dropout that did not impute. In particular, 11 out of the 16 (68.8*%*) papers with more than 30*%* of patients dropping out of the trial used no imputation methods. This is a potentially worryingly high figure, as failing to utilise the information provided by missing data, when there is such a large amount of missingness, can cause us to draw misleading conclusions. It has been suggested in the past that trials with over a 15*%* dropout rate are in need of the missing data to be analysed and addressed [2]. Also 30 (33.0%) papers used inappropriate missing data handling methods, and 13 (35.1%) of the papers that attempted to explain the reasons behind their choice of method provided an inadequate justification. For clarity and to provide maximum information, one suggested technique is to present the imputed dataset results alongside the results for just complete case analysis. This was done in 7 out of 18 (38.9*%*) papers with imputation methods used. Out of these 7 papers, 2 studies yielded different conclusions when some of the imputed results were compared to the complete case; both of these papers had over 30% dropout. One of the two papers in particular used a wide range of imputation methods, and a significant treatment effect was given when LOCF was used, although LOCF was not selected as an appropriate missing data handling method in this paper, and the clinical conclusions would have been the same based on the primary analysis compared with the complete case analysis. This highlights the need to proceed cautiously when choosing an appropriate missing data handling method. However, we acknowledge that it may be difficult to assess the magnitude of this problem from just two papers.

In such a widespread and financial-based industry, the idea that a clinical trial may provide inaccurate conclusions due to the failure to address missing data is a worrying thought. We must ask ourselves how we are going to prevent such an issue from occurring; something which the findings of this report indicate is failing to be addressed.

The discussions within this paper have been largely directed towards appropriate methods of statistical analysis, with a particular emphasis on the use of imputation. Missing data becomes less of a problem when we can obtain larger amounts of information on the patients that dropped out, as well as minimise the amount of missing data within a trial. When possible in trials with longitudinal measurements, clinicians and trialists should ensure that the trial design considers the potential for missing data arising in the study and aims to take precautions to try reduce the amount of missingness within a study.

### Guidelines for missing data handling: the four-point plan

The issue of missing data in general (not specific to longitudinal outcome data) is not dealt with in great detail in the CONSORT statement. It may be of use to suggest the following guidelines in order to formalise a procedure of missing data handling within longitudinal trials. This is done with the interest of ensuring we are obtaining accurate prognostic conclusions by not failing to recognise the problems that come with ignoring missing data within a trial. 

• Within a trial the reasons for missing data and, more specifically, the reasons for dropout, should be reported in detail. This can be defined as each individual patient’s reason for dropout being recorded within the study.

• After assessing these reasons for missingness, there should be detailed discussions as to the methods that will be used for missing data handling.

• These methods should then be justified within the report, and their potential limitations described.

• When the final analysis is carried out, any imputed dataset results should be presented alongside the complete case analysis results.

### Joint modelling and the MAGNETIC trial

**Figure 4:**
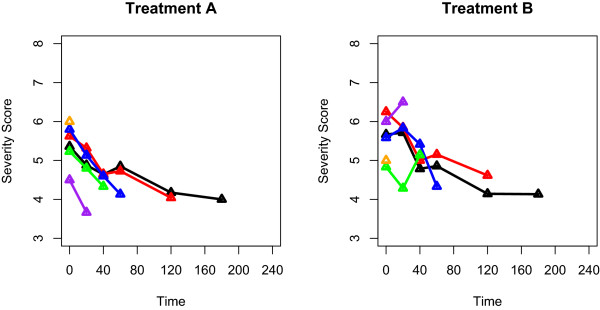
**Severity score profiles for MAGNETIC.** The mean severity score profiles for the patients who dropped out at each time point. The mean dropout at each time point corresponds to a different colour of line on the graph. The first panel represents the patients that were administered to Treatment **A**, and likewise the second panel for Treatment **B**.

For an example of how missing data could be handled in a trial, one could point in the direction of the recently published MAGNETIC trial [12]. This trial clearly stated its aims as well as the reasons why patients were dropped out. Once these reasons were assessed, suitable methods were chosen and results were presented for the complete case analysis; missing data methods were also used. One particular method employed in this trial, which was not used in any of the papers included in this systematic review, is ‘joint modelling of longitudinal and time-to-event data’. One benefit of using these joint models is that they are able to model the longitudinal outcome over time while accounting for the dropout. Within the MAGNETIC trial, a complete case analysis failed to show a difference between a placebo and the magnesium nebuliser treatment for reducing the severity of asthma. However, by employing joint modelling techniques they were able to establish a statistically significant difference in the success of the treatments due to the difference in mean dropout profiles for each treatment group (Figure 4).

By following the example of the MAGNETIC trial, and the four-point plan that we have proposed, we can ensure that we do not allow the missing data to be a catalyst for inaccurate conclusions within randomised control trials with longitudinal readings.

### Limitations

There is always a risk of bias when randomly selecting a subset of papers; however, we felt that this was a more informative method than selecting only papers from high impact journals. The possibility was considered that higher impact journals may publish trials with greater details of missing data. While little research has been done in to confirm or deny this, not putting a restriction on journal eliminated this potential problem. Should more time be available, it would be informative to assess the missing data mechanisms of all 381 papers that were regarded as eligible.

## Conclusions

This study indicates that a large proportion of papers failed to recognise the issue of missing data, or at best failed to give enough information in order to ensure that an accurate method of missing data handling was used. The majority of papers failed to explain their reasons for the method of missing data handling employed within their trial. In addition, less than 40% of papers gave detailed reasons for the missingness. Collecting the reasons for missing data can prove a valuable and important asset in order to establish the consistency of trials as well as draw accurate conclusions. Investigators should use these reasons for missingness in order to establish an appropriate method for missing data handling, and it is not necessarily the case that all dropouts within a trial should be subjected to the same imputation method, if imputation is used. There was very little consistency in the levels that the different trials used to consider the problems caused by missing data. In general, a greater awareness is needed in order to ensure that clinical investigators can obtain clinically accurate results from the trial in question by making informed choices and using appropriate methods of missing data handling.

## Abbreviations

CONSORT: Consolidated Standards of Reporting Trials; RCT: randomised controlled trial.

## Competing interests

The authors declare that they have no competing interests.

## Authors’ contributions

MP co-developed the protocol, developed the search strategy, carried out the search and data extraction, and drafted the manuscript as well as providing final approval of the version to be published. RKD co-developed the protocol, approved the search strategy, aided with any extracted data ambiguities and provided comments and assistance for the drafts of the manuscript, as well as providing final approval of the version to be published. JK co-developed the protocol, approved the search strategy, aided with any extracted data ambiguities and contributed to revisions of drafts of the manuscript as well as providing final approval of the version to be published. PW helped to conceive the initial idea, and provided comments and assistance throughout the study on both the extracted data and drafts of the manuscript, as well as providing final approval of the version to be published.

## Supplementary Material

Additional file 1 **Supplementary material.** Papers included in the systematic review. This is a document which lists the 100 papers that were included within our study. Click here for file
