# Supplementary material for: A review of the handling of missing longitudinal outcome data in clinical trials
Source: Trials. 2014 Jun 19;15:237. doi: 10.1186/1745-6215-15-237 (PMC4087243; doi:10.1186/1745-6215-15-237)
Supplement: Additional file 1 — Supplementary material. Papers included in the systematic review. This is a document which lists the 100 papers that were included within our study. [file 1745-6215-15-237-S1.doc]

Supplementary Material:

Papers included in the Systematic Review

1. Afshar H, Roohafza H, Mousavi G, Golchin S, Toghianifar N, Sadeghi M, Talaei M: **Topiramate add-on treatment in schizophrenia: a randomised, double-blind, placebo-controlled clinical trial**. *Journal of psychopharmacology* 2009, **23**(2):157-162.

2. Ambrosino JM, Fennie K, Whittemore R, Jaser S, Dowd MF, Grey M: **Short-term effects of coping skills training in school-age children with type 1 diabetes**. *Pediatric diabetes* 2008, **9**(3 Pt 2):74-82.

3. Arteaga-Troncoso G, Villegas-Alvarado A, Belmont-Gomez A, Martinez-Herrera FJ, Villagrana-Zesati R, Guerra-Infante F: **Intracervical application of the nitric oxide donor isosorbide dinitrate for induction of cervical ripening: a randomised controlled trial to determine clinical efficacy and safety prior to first trimester surgical evacuation of retained products of conception**. *BJOG : an international journal of obstetrics and gynaecology* 2005, **112**(12):1615-1619.

4. Berger AM, Kuhn BR, Farr LA, Lynch JC, Agrawal S, Chamberlain J, Von Essen SG: **Behavioral therapy intervention trial to improve sleep quality and cancer-related fatigue**. *Psycho-oncology* 2009, **18**(6):634-646.

5. Bird SE, Williams K, Kula K: **Preoperative acetaminophen vs ibuprofen for control of pain after orthodontic separator placement**. *American journal of orthodontics and dentofacial orthopedics : official publication of the American Association of Orthodontists, its constituent societies, and the American Board of Orthodontics* 2007, **132**(4):504-510.

6. Callahan LF, Mielenz T, Freburger J, Shreffler J, Hootman J, Brady T, Buysse K, Schwartz T: **A randomized controlled trial of the people with arthritis can exercise program: symptoms, function, physical activity, and psychosocial outcomes**. *Arthritis and rheumatism* 2008, **59**(1):92-101.

7. Chae J, Yu DT, Walker ME, Kirsteins A, Elovic EP, Flanagan SR, Harvey RL, Zorowitz RD, Frost FS, Grill JH *et al*: **Intramuscular Electrical Stimulation for Hemiplegic Shoulder Pain**. *American Journal of Physical Medicine & Rehabilitation* 2005, **84**(11):832-842.

8. Cortelli JR, Querido SM, Aquino DR, Ricardo LH, Pallos D: **Longitudinal clinical evaluation of adjunct minocycline in the treatment of chronic periodontitis**. *Journal of periodontology* 2006, **77**(2):161-166.

9. Doheny MO, Sedlak CA, Hall RJ, Estok PJ: **Structural model for osteoporosis preventing behavior in men**. *American journal of men's health* 2010, **4**(4):334-343.

10. Dorstyn DS, Mathias JL, Denson LA: **Psychological intervention during spinal rehabilitation: a preliminary study**. *Spinal cord* 2010, **48**(10):756-761.

11. Dougherty CM, Thompson EA, Lewis FM: **Long-term outcomes of a telephone intervention after an ICD**. *Pacing and clinical electrophysiology : PACE* 2005, **28**(11):1157-1167.

12. Elnour AA, El Mugammar IT, Jaber T, Revel T, McElnay JC: **Pharmaceutical care of patients with gestational diabetes mellitus**. *Journal of evaluation in clinical practice* 2008, **14**(1):131-140.

13. Estok PJ, Sedlak CA, Doheny MO, Hall R: **Structural model for osteoporosis preventing behavior in postmenopausal women**. *Nursing research* 2007, **56**(3):148-158.

14. Fader JP, Cleary RK, Lampman RM, Winter S, Singal BM, Plona AE: **Does intrathecal morphine sulfate provide preemptive analgesia for patients undergoing stapled hemorrhoidopexy?** *Pain medicine (Malden, Mass)* 2011, **12**(2):322-327.

15. Fernandez WG, Winter MR, Mitchell PM, Bullock H, Donovan J, St George J, Feldman JA, Gallagher SS, McKay MP, Bernstein E *et al*: **Six-month follow-up of a brief intervention on self-reported safety belt use among emergency department patients**. *Academic emergency medicine : official journal of the Society for Academic Emergency Medicine* 2009, **16**(11):1221-1224.

16. Fields C, Drye L, Vaidya V, Lyketsos C, Group AR: **Celecoxib or naproxen treatment does not benefit depressive symptoms in persons age 70 and older: findings from a randomized controlled trial**. *The American journal of geriatric psychiatry : official journal of the American Association for Geriatric Psychiatry* 2012, **20**(6):505-513.

17. Fortin PR, Abrahamowicz M, Ferland D, Lacaille D, Smith CD, Zummer M, Canadian Network For Improved Outcomes in Systemic L: **Steroid-sparing effects of methotrexate in systemic lupus erythematosus: a double-blind, randomized, placebo-controlled trial**. *Arthritis and rheumatism* 2008, **59**(12):1796-1804.

18. Frawley HC, Phillips BA, Bo K, Galea MP: **Physiotherapy as an adjunct to prolapse surgery: an assessor-blinded randomized controlled trial**. *Neurourology and urodynamics* 2010, **29**(5):719-725.

19. Friese RS, Barber R, McBride D, Bender J, Gentilello LM: **Could Beta blockade improve outcome after injury by modulating inflammatory profiles?** *The Journal of trauma* 2008, **64**(4):1061-1068.

20. Fukui S, Ogawa K, Yamagishi A: **Effectiveness of communication skills training of nurses on the quality of life and satisfaction with healthcare professionals among newly diagnosed cancer patients: a preliminary study**. *Psycho-oncology* 2011, **20**(12):1285-1291.

21. Garrett WE, Kaeding CC, ElAttrache NS, Xerogeanes JW, Hewitt MS, Skrepnik NV, Papilion JD, O'Donnell JB, Fox DL, Ruvuna F *et al*: **Novel drug OMS103HP reduces pain and improves joint motion and function for 90 days after arthroscopic meniscectomy**. *Arthroscopy : the journal of arthroscopic & related surgery : official publication of the Arthroscopy Association of North America and the International Arthroscopy Association* 2011, **27**(8):1060-1070.

22. Goldman RH, Stason WB, Park SK, Kim R, Mudgal S, Davis RB, Kaptchuk TJ: **Low-dose amitriptyline for treatment of persistent arm pain due to repetitive use**. *Pain* 2010, **149**(1):117-123.

23. Goodyer I, Dubicka B, Wilkinson P, Kelvin R, Roberts C, Byford S, Breen S, Ford C, Barrett B, Leech A *et al*: **Selective serotonin reuptake inhibitors (SSRIs) and routine specialist care with and without cognitive behaviour therapy in adolescents with major depression: randomised controlled trial**. *Bmj* 2007, **335**(7611):142.

24. Grandes G, Sanchez A, Montoya I, Ortega Sanchez-Pinilla R, Torcal J, Group P: **Two-year longitudinal analysis of a cluster randomized trial of physical activity promotion by general practitioners**. *PloS one* 2011, **6**(3):e18363.

25. Gustavsson C, Denison E, von Koch L: **Self-management of persistent neck pain: two-year follow-up of a randomized controlled trial of a multicomponent group intervention in primary health care**. *Spine* 2011, **36**(25):2105-2115.

26. Hansen NB, Tarakeshwar N, Ghebremichael M, Zhang H, Kochman A, Sikkema KJ: **Longitudinal effects of coping on outcome in a randomized controlled trial of a group intervention for HIV-positive adults with AIDS-related bereavement**. *Death studies* 2006, **30**(7):609-636.

27. Harrington JW, Logan S, Harwell C, Gardner J, Swingle J, McGuire E, Santos R: **Effective analgesia using physical interventions for infant immunizations**. *Pediatrics* 2012, **129**(5):815-822.

28. Hatfield LA: **Sucrose decreases infant biobehavioral pain response to immunizations: a randomized controlled trial**. *Journal of nursing scholarship : an official publication of Sigma Theta Tau International Honor Society of Nursing / Sigma Theta Tau* 2008, **40**(3):219-225.

29. Hatfield LA, Gusic ME, Dyer AM, Polomano RC: **Analgesic properties of oral sucrose during routine immunizations at 2 and 4 months of age**. *Pediatrics* 2008, **121**(2):e327-334.

30. Heiney SP, Millon Underwood S, Tavakoli A, Arp Adams S, Wells LM, Bryant LH: **Randomized trial of therapeutic group by teleconference: African American women with breast cancer**. *Cancer* 2012, **118**(15):3822-3832.

31. Holmes-Rovner M, Stommel M, Corser WD, Olomu A, Holtrop JS, Siddiqi A, Dunn SL: **Does outpatient telephone coaching add to hospital quality improvement following hospitalization for acute coronary syndrome?** *Journal of general internal medicine* 2008, **23**(9):1464-1470.

32. Hovell MF, Nichols JF, Irvin VL, Schmitz KE, Rock CL, Hofstetter CR, Keating K, Stark LJ: **Parent/Child training to increase preteens' calcium, physical activity, and bone density: a controlled trial**. *American journal of health promotion : AJHP* 2009, **24**(2):118-128.

33. Howell EA, Balbierz A, Wang J, Parides M, Zlotnick C, Leventhal H: **Reducing postpartum depressive symptoms among black and Latina mothers: a randomized controlled trial**. *Obstetrics and gynecology* 2012, **119**(5):942-949.

34. Husebo BS, Ballard C, Sandvik R, Nilsen OB, Aarsland D: **Efficacy of treating pain to reduce behavioural disturbances in residents of nursing homes with dementia: cluster randomised clinical trial**. *Bmj* 2011, **343**:d4065.

35. Iannotti LL, Zavaleta N, Leon Z, Shankar AH, Caulfield LE: **Maternal zinc supplementation and growth in Peruvian infants**. *The American journal of clinical nutrition* 2008, **88**(1):154-160.

36. James J, Thomas P, Kerr D: **Preventing childhood obesity: two year follow-up results from the Christchurch obesity prevention programme in schools (CHOPPS)**. *Bmj* 2007, **335**(7623):762.

37. Jenkins AL, Kacinik V, Lyon M, Wolever TM: **Effect of adding the novel fiber, PGX(R), to commonly consumed foods on glycemic response, glycemic index and GRIP: a simple and effective strategy for reducing post prandial blood glucose levels--a randomized, controlled trial**. *Nutrition journal* 2010, **9**:58.

38. Kaisar MO, Wiggins KJ, Sturtevant JM, Hawley CM, Campbell SB, Isbel NM, Mudge DW, Bofinger A, Petrie JJ, Johnson DW: **A randomized controlled trial of fludrocortisone for the treatment of hyperkalemia in hemodialysis patients**. *American journal of kidney diseases : the official journal of the National Kidney Foundation* 2006, **47**(5):809-814.

39. Kaplan K, Salzer MS, Solomon P, Brusilovskiy E, Cousounis P: **Internet peer support for individuals with psychiatric disabilities: A randomized controlled trial**. *Social science & medicine* 2011, **72**(1):54-62.

40. Katrak S, Gasasira A, Arinaitwe E, Kakuru A, Wanzira H, Bigira V, Sandison TG, Homsy J, Tappero JW, Kamya MR *et al*: **Safety and tolerability of artemether-lumefantrine versus dihydroartemisinin-piperaquine for malaria in young HIV-infected and uninfected children**. *Malaria journal* 2009, **8**:272.

41. Keefe MR, Kajrlsen KA, Lobo ML, Kotzer AM, Dudley WN: **Reducing parenting stress in families with irritable infants**. *Nursing research* 2006, **55**(3):198-205.

42. Kendall E, Catalano T, Kuipers P, Posner N, Buys N, Charker J: **Recovery following stroke: the role of self-management education**. *Social science & medicine* 2007, **64**(3):735-746.

43. Klehmet J, Harms H, Richter M, Prass K, Volk HD, Dirnagl U, Meisel A, Meisel C: **Stroke-induced immunodepression and post-stroke infections: lessons from the preventive antibacterial therapy in stroke trial**. *Neuroscience* 2009, **158**(3):1184-1193.

44. Kolls BJ, Stacy M: **Apomorphine**. *Clinical Neuropharmacology* 2006, **29**(5):292-301.

45. Kravitz RL, Tancredi DJ, Jerant A, Saito N, Street RL, Grennan T, Franks P: **Influence of patient coaching on analgesic treatment adjustment: secondary analysis of a randomized controlled trial**. *Journal of pain and symptom management* 2012, **43**(5):874-884.

46. Kuhnle MD, Ryan DS, Coe CD, Eaddy J, Kuzmowych C, Edwards J, Howard RS, Bower KS: **Oral gabapentin for photorefractive keratectomy pain**. *Journal of cataract and refractive surgery* 2011, **37**(2):364-369.

47. Lai HL, Hwang MJ, Chen CJ, Chang KF, Peng TC, Chang FM: **Randomised controlled trial of music on state anxiety and physiological indices in patients undergoing root canal treatment**. *Journal of clinical nursing* 2008, **17**(19):2654-2660.

48. Langhammer B, Stanghelle JK, Lindmark B: **An evaluation of two different exercise regimes during the first year following stroke: a randomised controlled trial**. *Physiotherapy theory and practice* 2009, **25**(2):55-68.

49. Lara MA, Navarro C, Navarrete L: **Outcome results of a psycho-educational intervention in pregnancy to prevent PPD: a randomized control trial**. *Journal of affective disorders* 2010, **122**(1-2):109-117.

50. Lee JT, Yen HW: **Randomized controlled evaluation of a theory-based postpartum sexual health education programme**. *Journal of advanced nursing* 2007, **60**(4):389-401.

51. Liedl A, Muller J, Morina N, Karl A, Denke C, Knaevelsrud C: **Physical activity within a CBT intervention improves coping with pain in traumatized refugees: results of a randomized controlled design**. *Pain medicine (Malden, Mass)* 2011, **12**(2):234-245.

52. Lin PH, Appel LJ, Funk K, Craddick S, Chen C, Elmer P, McBurnie MA, Champagne C: **The PREMIER intervention helps participants follow the Dietary Approaches to Stop Hypertension dietary pattern and the current Dietary Reference Intakes recommendations**. *Journal of the American Dietetic Association* 2007, **107**(9):1541-1551.

53. Lo C, Burman D, Hales S, Swami N, Rodin G, Zimmermann C: **The FAMCARE-Patient scale: measuring satisfaction with care of outpatients with advanced cancer**. *European journal of cancer* 2009, **45**(18):3182-3188.

54. Lorenzo R, Garcia V, Orsini M, Martin C, Sanz M: **Clinical efficacy of a xenogeneic collagen matrix in augmenting keratinized mucosa around implants: a randomized controlled prospective clinical trial**. *Clinical oral implants research* 2012, **23**(3):316-324.

55. Lovell DJ, Glass D, Ranz J, Kramer S, Huang B, Sierra RI, Henderson CJ, Passo M, Graham B, Bowyer S *et al*: **A randomized controlled trial of calcium supplementation to increase bone mineral density in children with juvenile rheumatoid arthritis**. *Arthritis and rheumatism* 2006, **54**(7):2235-2242.

56. Marcantonio ER, Palihnich K, Appleton P, Davis RB: **Pilot randomized trial of donepezil hydrochloride for delirium after hip fracture**. *Journal of the American Geriatrics Society* 2011, **59 Suppl 2**:S282-288.

57. Markland AD, Richter HE, Burgio KL, Myers DL, Hernandez AL, Subak LL: **Weight loss improves fecal incontinence severity in overweight and obese women with urinary incontinence**. *International urogynecology journal* 2011, **22**(9):1151-1157.

58. Marr M, Baker J, Lambon N, Perry J: **The effects of the Bowen technique on hamstring flexibility over time: a randomised controlled trial**. *Journal of bodywork and movement therapies* 2011, **15**(3):281-290.

59. Maturi MS, Afshary P, Abedi P: **Effect of physical activity intervention based on a pedometer on physical activity level and anthropometric measures after childbirth: a randomized controlled trial**. *BMC pregnancy and childbirth* 2011, **11**:103.

60. McCurry SM, Gibbons LE, Logsdon RG, Vitiello MV, Teri L: **Nighttime insomnia treatment and education for Alzheimer's disease: a randomized, controlled trial**. *Journal of the American Geriatrics Society* 2005, **53**(5):793-802.

61. McKinley S, Dracup K, Moser DK, Riegel B, Doering LV, Meischke H, Aitken LM, Buckley T, Marshall A, Pelter M: **The effect of a short one-on-one nursing intervention on knowledge, attitudes and beliefs related to response to acute coronary syndrome in people with coronary heart disease: a randomized controlled trial**. *International journal of nursing studies* 2009, **46**(8):1037-1046.

62. McMurray JS, Connor N, Ford CN: **Cidofovir efficacy in recurrent respiratory papillomatosis: a randomized, double-blind, placebo-controlled study**. *The Annals of otology, rhinology, and laryngology* 2008, **117**(7):477-483.

63. Meneses K, McNees P, Azuero A, Loerzel VW, Su X, Hassey LA: **Preliminary evaluation of psychoeducational support interventions on quality of life in rural breast cancer survivors after primary treatment**. *Cancer nursing* 2009, **32**(5):385-397.

64. Miyashita M: **Effects of continuous versus accumulated activity patterns on postprandial triacylglycerol concentrations in obese men**. *International journal of obesity* 2008, **32**(8):1271-1278.

65. Naar-King S, Parsons JT, Murphy DA, Chen X, Harris DR, Belzer ME: **Improving health outcomes for youth living with the human immunodeficiency virus: a multisite randomized trial of a motivational intervention targeting multiple risk behaviors**. *Archives of pediatrics & adolescent medicine* 2009, **163**(12):1092-1098.

66. Nabulsi MM, Tamim H, Mahfoud Z, Itani M, Sabra R, Chamseddine F, Mikati M: **Alternating ibuprofen and acetaminophen in the treatment of febrile children: a pilot study [ISRCTN30487061]**. *BMC medicine* 2006, **4**:4.

67. Neiva RF, Al-Shammari K, Nociti FH, Jr., Soehren S, Wang HL: **Effects of vitamin-B complex supplementation on periodontal wound healing**. *Journal of periodontology* 2005, **76**(7):1084-1091.

68. Orrell M, Spector A, Thorgrimsen L, Woods B: **A pilot study examining the effectiveness of maintenance Cognitive Stimulation Therapy (MCST) for people with dementia**. *International journal of geriatric psychiatry* 2005, **20**(5):446-451.

69. Pijls BG, Valstar ER, Kaptein BL, Fiocco M, Nelissen RG: **The beneficial effect of hydroxyapatite lasts: a randomized radiostereometric trial comparing hydroxyapatite-coated, uncoated, and cemented tibial components for up to 16 years**. *Acta orthopaedica* 2012, **83**(2):135-141.

70. Poole H, Glenn S, Murphy P: **A randomised controlled study of reflexology for the management of chronic low back pain**. *European journal of pain* 2007, **11**(8):878-887.

71. Potterton J, Stewart A, Cooper P, Becker P: **The effect of a basic home stimulation programme on the development of young children infected with HIV**. *Developmental medicine and child neurology* 2010, **52**(6):547-551.

72. Power MJ, Freeman C: **A randomized controlled trial of IPT versus CBT in primary care: with some cautionary notes about handling missing values in clinical trials**. *Clinical psychology & psychotherapy* 2012, **19**(2):159-169.

73. Pradhan EK, Baumgarten M, Langenberg P, Handwerger B, Gilpin AK, Magyari T, Hochberg MC, Berman BM: **Effect of Mindfulness-Based Stress Reduction in rheumatoid arthritis patients**. *Arthritis and rheumatism* 2007, **57**(7):1134-1142.

74. Pritchett Y, Jemiai Y, Chang Y, Bhan I, Agarwal R, Zoccali C, Wanner C, Lloyd-Jones D, Cannata-Andia JB, Thompson T *et al*: **The use of group sequential, information-based sample size re-estimation in the design of the PRIMO study of chronic kidney disease**. *Clinical trials* 2011, **8**(2):165-174.

75. Pulido JS, Winters JL, Boyer D: **Preliminary analysis of the final multicenter investigation of rheopheresis for age related macular degeneration (AMD) trial (MIRA-1) results**. *Transactions of the American Ophthalmological Society* 2006, **104**:221-231.

76. Richardson LC, Wang W, Hartzema AG, Wagner S: **The role of health-related quality of life in early discontinuation of chemotherapy for breast cancer**. *The breast journal* 2007, **13**(6):581-587.

77. Robinson P, Keenan AM, Conaghan PG: **Clinical effectiveness and dose response of image-guided intra-articular corticosteroid injection for hip osteoarthritis**. *Rheumatology* 2007, **46**(2):285-291.

78. Romieu I, Riojas-Rodriguez H, Marron-Mares AT, Schilmann A, Perez-Padilla R, Masera O: **Improved biomass stove intervention in rural Mexico: impact on the respiratory health of women**. *American journal of respiratory and critical care medicine* 2009, **180**(7):649-656.

79. Rostami HR, Malamiri RA: **Effect of treatment environment on modified constraint-induced movement therapy results in children with spastic hemiplegic cerebral palsy: a randomized controlled trial**. *Disability and rehabilitation* 2012, **34**(1):40-44.

80. Sadik A, Yousif M, McElnay JC: **Pharmaceutical care of patients with heart failure**. *British journal of clinical pharmacology* 2005, **60**(2):183-193.

81. Sanz M, Lorenzo R, Aranda JJ, Martin C, Orsini M: **Clinical evaluation of a new collagen matrix (Mucograft prototype) to enhance the width of keratinized tissue in patients with fixed prosthetic restorations: a randomized prospective clinical trial**. *Journal of clinical periodontology* 2009, **36**(10):868-876.

82. Shiga T, Yamada Y, Matsuda N, Tanaka T, Urae A, Hashiguchi M, Hagiwara N, Kasanuki H: **Influence of cilnidipine or nisoldipine on sympathetic activity in healthy male subjects**. *Heart and vessels* 2007, **22**(6):404-409.

83. Shim JC, Jung DU, Jung SS, Seo YS, Cho DM, Lee JH, Lee SW, Kong BG, Kang JW, Oh MK *et al*: **Adjunctive varenicline treatment with antipsychotic medications for cognitive impairments in people with schizophrenia: a randomized double-blind placebo-controlled trial**. *Neuropsychopharmacology : official publication of the American College of Neuropsychopharmacology* 2012, **37**(3):660-668.

84. Shiroiwa T, Fukuda T, Shimozuma K, Kuranami M, Suemasu K, Ohashi Y, Watanabe T: **Comparison of EQ-5D scores among anthracycline-containing regimens followed by taxane and taxane-only regimens for node-positive breast cancer patients after surgery: the N-SAS BC 02 trial**. *Value in health : the journal of the International Society for Pharmacoeconomics and Outcomes Research* 2011, **14**(5):746-751.

85. Song L, Northouse LL, Braun TM, Zhang L, Cimprich B, Ronis DL, Mood DW: **Assessing longitudinal quality of life in prostate cancer patients and their spouses: a multilevel modeling approach**. *Quality of life research : an international journal of quality of life aspects of treatment, care and rehabilitation* 2011, **20**(3):371-381.

86. Song L, Northouse LL, Zhang L, Braun TM, Cimprich B, Ronis DL, Mood DW: **Study of dyadic communication in couples managing prostate cancer: a longitudinal perspective**. *Psycho-oncology* 2012, **21**(1):72-81.

87. Stanton B, Guo J, Cottrell L, Galbraith J, Li X, Gibson C, Pack R, Cole M, Marshall S, Harris C: **The complex business of adapting effective interventions to new populations: an urban to rural transfer**. *The Journal of adolescent health : official publication of the Society for Adolescent Medicine* 2005, **37**(2):163.

88. Sun JL, Sung MS, Huang MY, Cheng GC, Lin CC: **Effectiveness of acupressure for residents of long-term care facilities with insomnia: a randomized controlled trial**. *International journal of nursing studies* 2010, **47**(7):798-805.

89. Swanenburg J, de Bruin ED, Stauffacher M, Mulder T, Uebelhart D: **Effects of exercise and nutrition on postural balance and risk of falling in elderly people with decreased bone mineral density: randomized controlled trial pilot study**. *Clinical rehabilitation* 2007, **21**(6):523-534.

90. Thorsdottir I, Tomasson H, Gunnarsdottir I, Gisladottir E, Kiely M, Parra MD, Bandarra NM, Schaafsma G, Martinez JA: **Randomized trial of weight-loss-diets for young adults varying in fish and fish oil content**. *International journal of obesity* 2007, **31**(10):1560-1566.

91. Tiersky LA, Anselmi V, Johnston MV, Kurtyka J, Roosen E, Schwartz T, Deluca J: **A trial of neuropsychologic rehabilitation in mild-spectrum traumatic brain injury**. *Archives of physical medicine and rehabilitation* 2005, **86**(8):1565-1574.

92. Trappenburg JC, Monninkhof EM, Bourbeau J, Troosters T, Schrijvers AJ, Verheij TJ, Lammers JW: **Effect of an action plan with ongoing support by a case manager on exacerbation-related outcome in patients with COPD: a multicentre randomised controlled trial**. *Thorax* 2011, **66**(11):977-984.

93. Tsui JI, Herman DS, Kettavong M, Anderson BJ, Stein MD: **Escitalopram is associated with reductions in pain severity and pain interference in opioid dependent patients with depressive symptoms**. *Pain* 2011, **152**(11):2640-2644.

94. Urech C, Fink NS, Hoesli I, Wilhelm FH, Bitzer J, Alder J: **Effects of relaxation on psychobiological wellbeing during pregnancy: a randomized controlled trial**. *Psychoneuroendocrinology* 2010, **35**(9):1348-1355.

95. Vempati R, Bijlani RL, Deepak KK: **The efficacy of a comprehensive lifestyle modification programme based on yoga in the management of bronchial asthma: a randomized controlled trial**. *BMC pulmonary medicine* 2009, **9**:37.

96. Venzke L, Calvert JF, Jr., Gilbertson B: **A randomized trial of acupuncture for vasomotor symptoms in post-menopausal women**. *Complementary therapies in medicine* 2010, **18**(2):59-66.

97. Vigano A, Giacomet V, Pariani E, Giani E, Manfredini V, Bedogni G, Erba P, Amendola A, Zanetti A, Zuccotti G: **Long-term immunogenicity after one and two doses of a monovalent MF59-adjuvanted A/H1N1 Influenza virus vaccine coadministered with the seasonal 2009-2010 nonadjuvanted Influenza virus vaccine in HIV-infected children, adolescents, and young adults in a randomized controlled trial**. *Clinical and vaccine immunology : CVI* 2011, **18**(9):1503-1509.

98. Wang TJ, Belza B, Elaine Thompson F, Whitney JD, Bennett K: **Effects of aquatic exercise on flexibility, strength and aerobic fitness in adults with osteoarthritis of the hip or knee**. *Journal of advanced nursing* 2007, **57**(2):141-152.

99. Whiteley P, Haracopos D, Knivsberg AM, Reichelt KL, Parlar S, Jacobsen J, Seim A, Pedersen L, Schondel M, Shattock P: **The ScanBrit randomised, controlled, single-blind study of a gluten- and casein-free dietary intervention for children with autism spectrum disorders**. *Nutritional neuroscience* 2010, **13**(2):87-100.

100. Yang XL, Li HH, Hong MH, Kao HS: **The effects of Chinese calligraphy handwriting and relaxation training in Chinese Nasopharyngeal Carcinoma patients: a randomized controlled trial**. *International journal of nursing studies* 2010, **47**(5):550-559.
